# Supplementary material for: A flexible catheter-based sensor array for upper airway soft tissues pressure monitoring
Source: Nat Commun. 2025 Jan 2;16:287. doi: 10.1038/s41467-024-55088-y (PMC11695590; doi:10.1038/s41467-024-55088-y)
Supplement: Supplementary file 1 — Supplementary Information [file 41467_2024_55088_MOESM1_ESM.pdf]

Supplementary Information for

A Flexible Catheter-Based Sensor Array for Upper Airway Soft Tissue Pressure Monitoring

Jiang Shang†, Xiaoxiao Ma†, Peikai Zou, Chenxiao Huang, Zhechen Lao, Junhan Wang, Tingshu Jiang, Yanzhe Fu, Jiebo Li, Shaoxing Zhang, Ruya Li*, Yubo Fan*

*Corresponding author:
Ruay Li, liruya@buaa.edu.cn; Yubo Fan, yubofan@buaa.edu.cn

This file includes:

Supplementary Text
Supplementary Figs. 1 to 24
Supplementary Tables 1 to 2

Supplementary Text

Supplementary Note S1: Derivation and simplified electrical model of the flexible piezoresistive sensor

Each sensing unit's resistance (R) comprises three components: the conductive layer's resistance on the PDMS membrane (R_{F1}), the conductive layer's resistance on the flexible catheter base microstructure (R_{F2}), and the conductive layers' contact resistance (R_C). When measuring contact pressure, the sensor experiences external pressure towards the center of the catheter. This pressure causes the PDMS membrane to deform, which in turn changes the contact area between the conductive layers on the PDMS membrane and the microstructures. As a result, the sensor's resistance is significantly altered. The sensor's resistance (R) as shown in：

$$R = R_{F1} + \frac{1/n \cdot R_C R_{F2}}{1/n \cdot R_C + R_{F2}}$$

With R representing the total sensor resistance, R_{F1} and R_{F2} representing the conductive layers' respective resistances, RC is the contact resistance, and n is the number of contact resistances. Following the resistance law $R=\rho L/S$, the resistance formula can be simplified as:

$$R = R_{F1} + a\frac{1}{S_C + b}$$

Where S_C is the contact area between the conductive layers on the PDMS membrane and the microstructures, $a = \rho_C d_C/n$, and $b = \rho_C d_C/nR_{F2}$. ρ_C and d_C are the resistivity and length of contact resistances (i.e. the resistivity and summed thicknesses of the conductive layers), and n is the number of contact resistances. ρ_C, d_C, and n all remain nearly constant. As the resistivity, length, and cross-sectional area of conductive layers on the catheter microstructures and PDMS membrane undergo minimal changes in deformation during pressure variation, the components R_{F1} and R_{F2} remain approximately constant under pressure, which makes a and b in equation constants. The contact area S_C between the conductive layers is the only variable. The sensor resistance output changes drastically with changes in pressure. An increase in pressure leads to an increase in S_C, which decreases the sensor's resistance. Similarly, a decrease in pressure causes the S_C to decrease, increasing the sensor's resistance.

Supplementary Note S2: bending modulus and Tensile Modulus test

Sample Preparation
The tubular samples used in this study were prepared in uniform lengths of 60 mm for tensile and bending tests to ensure consistent measurements.

Bending Modulus Measurement
Test setup: Bending tests were performed using a mechanical testing machine (EZ-LX HS, Shimadzu, Japan), configured with a three-point bending fixture (Supplementary Fig. 24a). The support span distance between the two supporting pins was set to 30 mm. The displacement of the midpoint of the sample was 10mm.

Procedure: Tubular samples were placed horizontally on the two supports, and a downward force was applied at the midpoint of the span. The test was conducted at a crosshead speed of 5 mm/min. Force and displacement data were recorded.

Bending Modulus Calculation: The bending modulus was calculated using the following equation:

$$\mathrm{E} = \frac{L^3}{48I} \times \frac{F}{\delta}$$

Where E is the bending modulus, *L* is the displacement of the midpoint (30 mm), *F* is the applied force, *δ* is the deflection at the midpoint, *I* is the second moment of the area of the tube, calculated as

$$I = \frac{\pi}{64} \times \left(D^4 - d^4\right)$$

where *D* is the outer diameter and *d* is the inner diameter. The bending modulus was derived from the slope of the initial linear portion of the force-displacement curve using linear regression of three repeated tests.

Tensile Modulus Measurement

Test setup: Tensile tests were conducted using a mechanical testing machine (EZ-LX HS, Shimadzu, Japan) (Supplementary Fig. 24b). The machine was equipped with standard tensile grips to securely hold the tubular sample ends. The tests were performed at a room temperature of 23°C under a constant strain rate of 5 mm/min.

Procedure: The test began by applying a uniaxial tensile load to the sample. The force and corresponding elongation were recorded continuously throughout the test.

Tensile Modulus Calculation: The force-extension data was converted to stress-strain data, using the following relationships:

$$\sigma = \frac{F}{A}$$

Where *σ* is the tensile stress, *F* is the force applied, *A* is the cross-sectional area of the tube, calculated as

$$I = \frac{\pi \times \left(D^2 - d^2\right)}{64}$$

Where *D* is the outer diameter, and *d* is the inner diameter. The tensile modulus was calculated from the initial linear portion of the stress-strain curve, the elastic region, using linear regression of three repeated tests.

<u>Supplementary Note S3: Establishment of the OSA Bama Pig Model</u>

Filling Mixture Composition and Injection

Under sedation, the bilateral palatal areas were topically anesthetized with lidocaine gel to ensure surface anesthesia. Injections were precisely administered at four sites on the palatopharyngeal

region of the model pigs (Supplementary Fig. 23). The central two sites were positioned 2cm away from the midline of the palate on both sides and approximately 3cm from the posterior edge of the hard palate. The remaining two sites were located on the lateral pharyngeal wall, posterior to the bilateral maxillary tubercles, parallel to the central sites. Before the injection, the preoperative CT helps determine the thickness of the soft palate, and the injection site is located at half the thickness of the soft palate. The filling mixture consisted of a combination of:

- crosslinked sodium hyaluronate gel (2ml)
- iohexol (1ml)
- penicillin injection solution (0.5ml at 80000U)
- iodoform (0.1g)

Injection Monitoring and Modeling Success Criteria

Injections were repeated until the animals displayed typical snoring symptoms and OSA characteristic events were observed on PSG. All animals were ultimately diagnosed with OSA based on clinical signs, CT scans, and PSG data. After successful modeling, PSG monitoring should be continued for more than 2 hours to ensure a stable and reliable animal model.

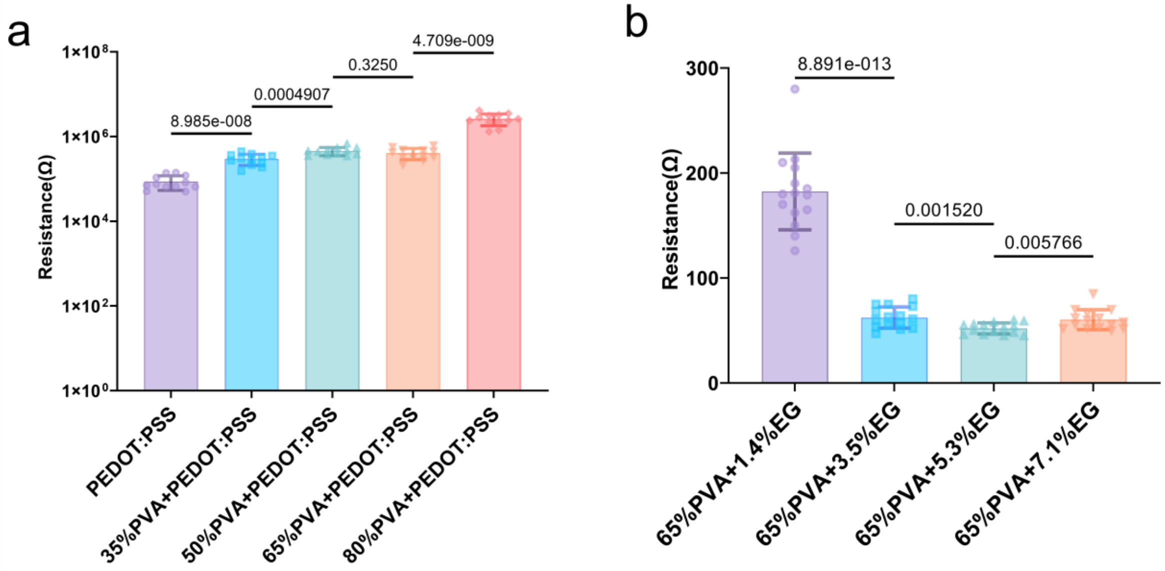

Supplementary Fig. 1. The impact of varying proportions of PVA and EG on the conductivity of the conductive layer. a The impact of PVA doping in PEDOT: PSS on the conductivity of the conductive layer. **b** The impact of varying proportions of EG doping in PEDOT: PSS, with a 65wt% PVA content, on the conductivity of the conductive layer. n=15, including 5 samples, each sample detecting 3 positions. Data are mean ± standard deviation, and comparisons between groups were made using the independent samples t-test.

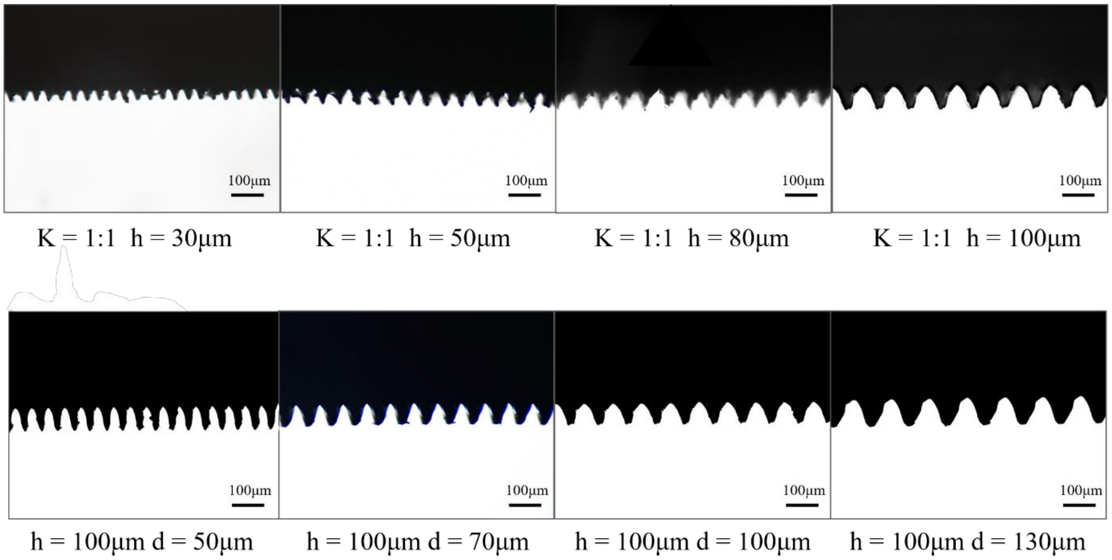

Supplementary Fig. 2. Metallographic microscope images of microstructures with different size parameters. Scale bar= 100μm.

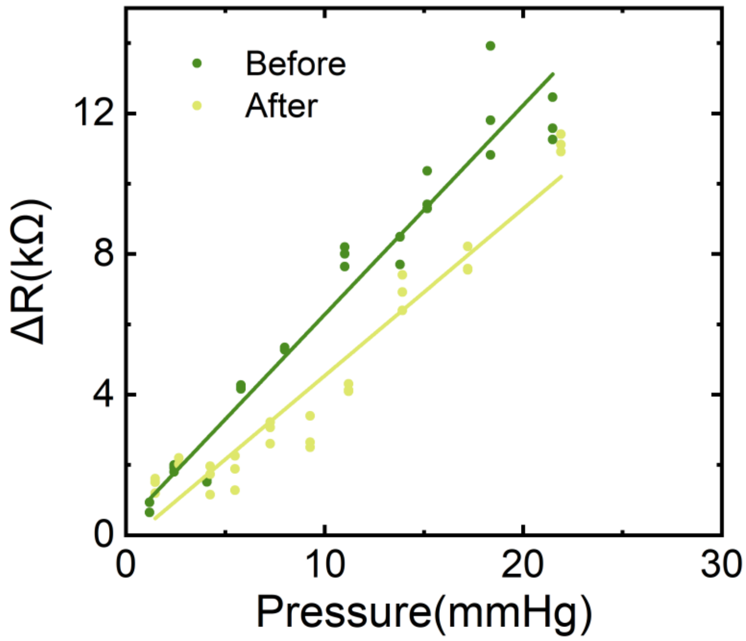

Supplementary Fig. 3. Sensitivity curves from the waterproofing test of the sensor.

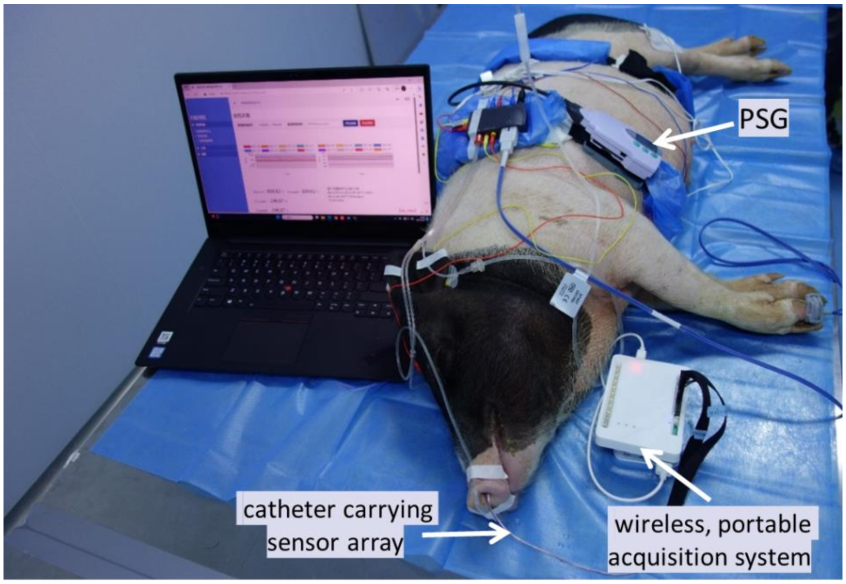

Supplementary Fig. 4. The catheter-based pressure sensor array and PSG were used to monitor the OSA model pig during sedation-induced sleep.

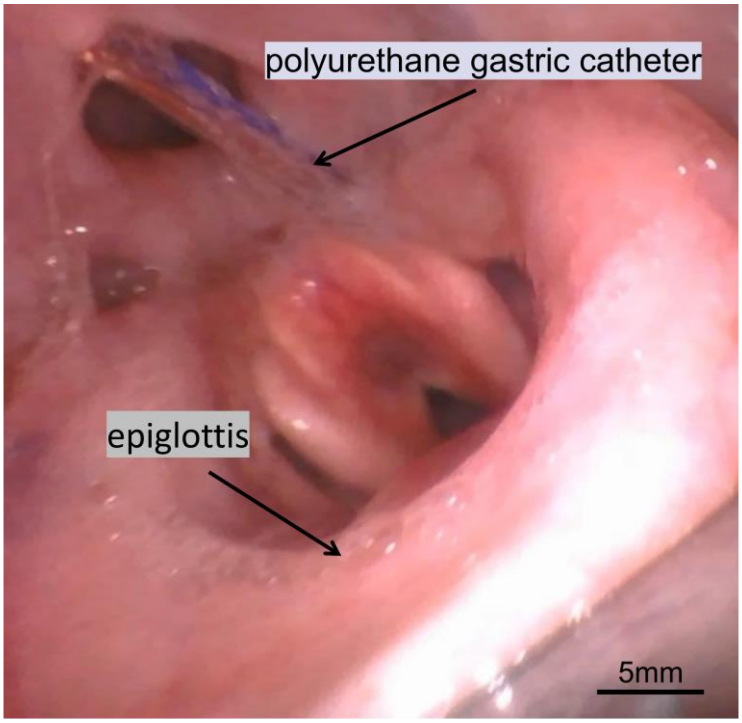

Supplementary Fig. 5. The endoscopic images revealed the catheter with the sensor array stopped at the epiglottis.

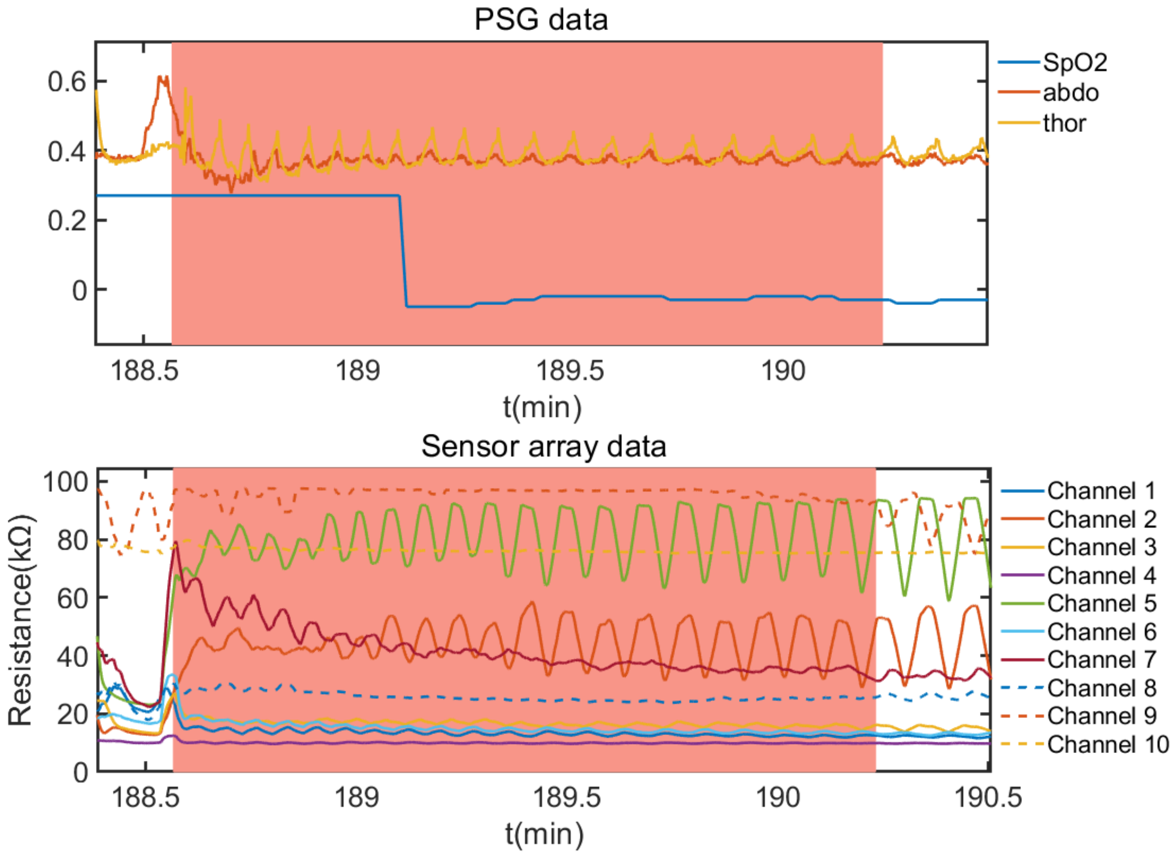

Supplementary Fig. 6. Corresponding PSG and sensor array data during snoring in OSA model pigs

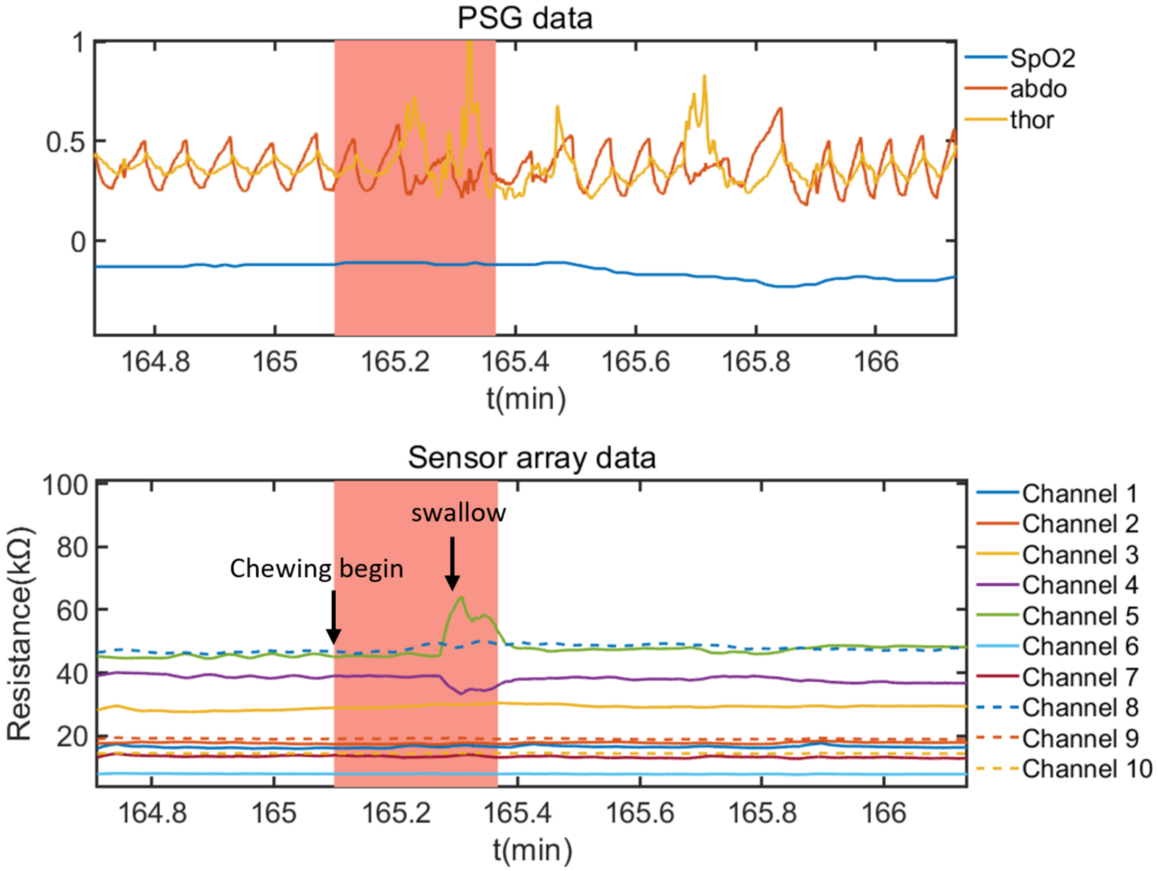

Supplementary Fig. 7. Corresponding data from PSG and sensor arrays during chewing and swallowing events in OSA model pigs.

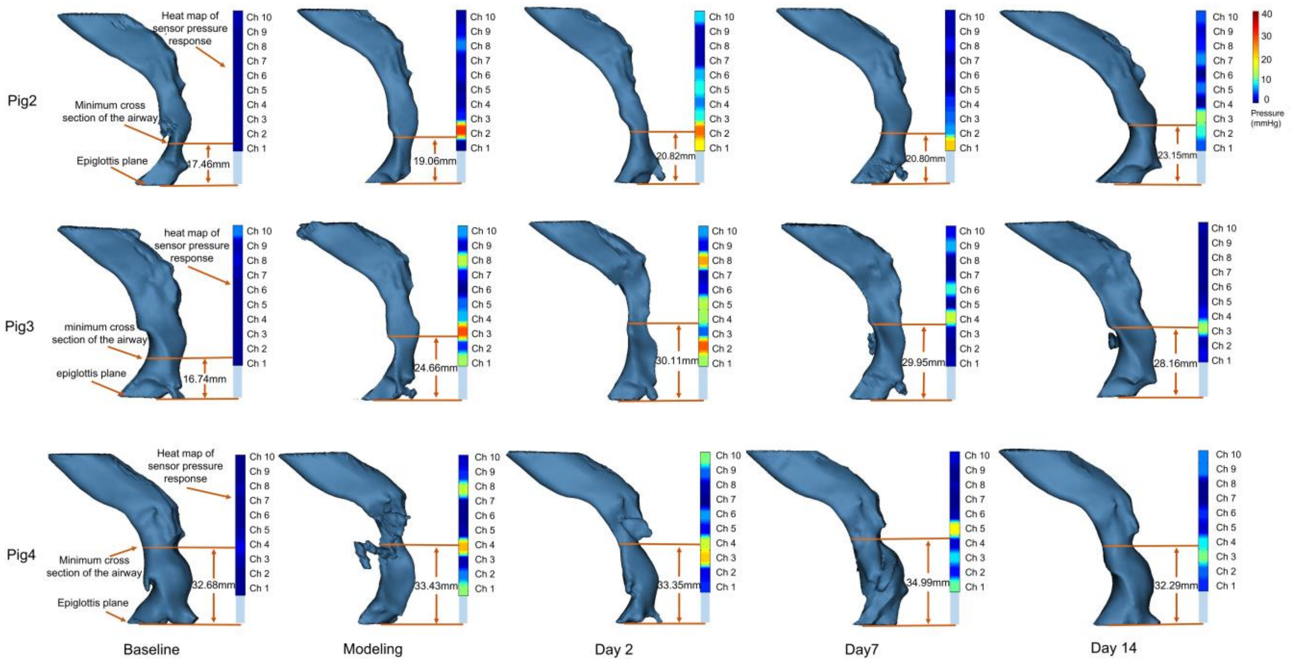

Supplementary Fig. 8. The position of the minimum cross-sectional area relative to the epiglottal plane in the upper airway CT images of the three OSA model pigs in the experimental group at different time points before and after modeling, and the heat map of the pressure response measured by the sensor array during OSA events.

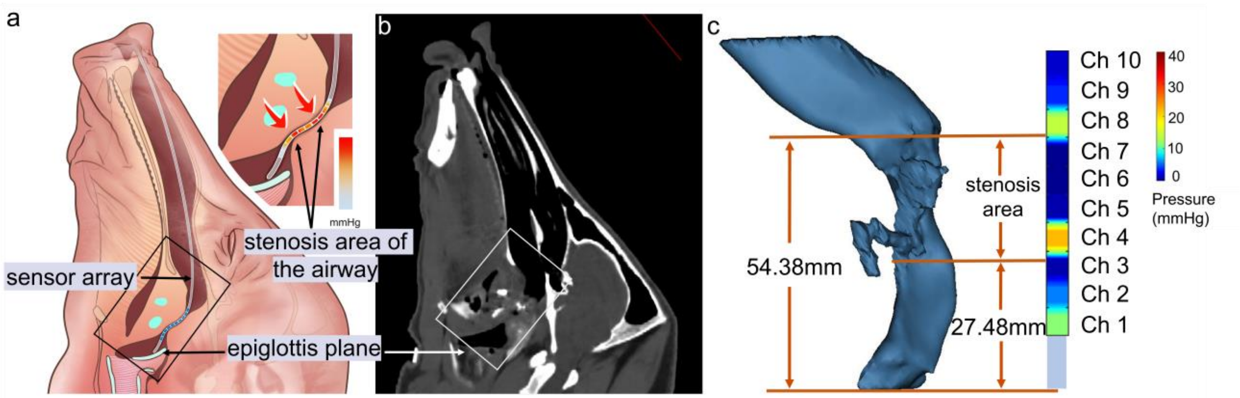

Supplementary Fig. 9 The application scenarios of the sensor array (a), upper airway CT image (b), and sensor pressure distribution heat map (c) in No.4 OSA model pig on the day of modeling. The location of the stenosis area in the upper airway CT reconstruction model was compared to the pressure distribution heatmap generated by the sensor array, with the epiglottis serving as the reference plane (**c**). On the day of modeling, CT imaging revealed two distinct stenotic areas within the airway, exhibiting a "double peak" characteristic with a total length of 26.9 mm (**b, c**). The heat map generated by the sensor array on this day demonstrated high contact pressure values for Ch4 and Ch8, with local pressures exceeding 15mmHg, while other sensor pressures remained below 10mmHg. Based on sensor unit distribution, Ch4 covered a sensing range from 30 to 36mm from the front of the catheter, whereas Ch8 had a sensing range from 54 to 60mm. Consequently, Ch4 to Ch8 collectively covered a total sensing area length of approximately 30mm. These measurement results confirmed that both location and length characteristics observed through CT imaging corresponded with pressure distribution depicted by the sensor array's heat map, thus validating our system's ability to detect multiple simultaneous airway obstructions accurately.

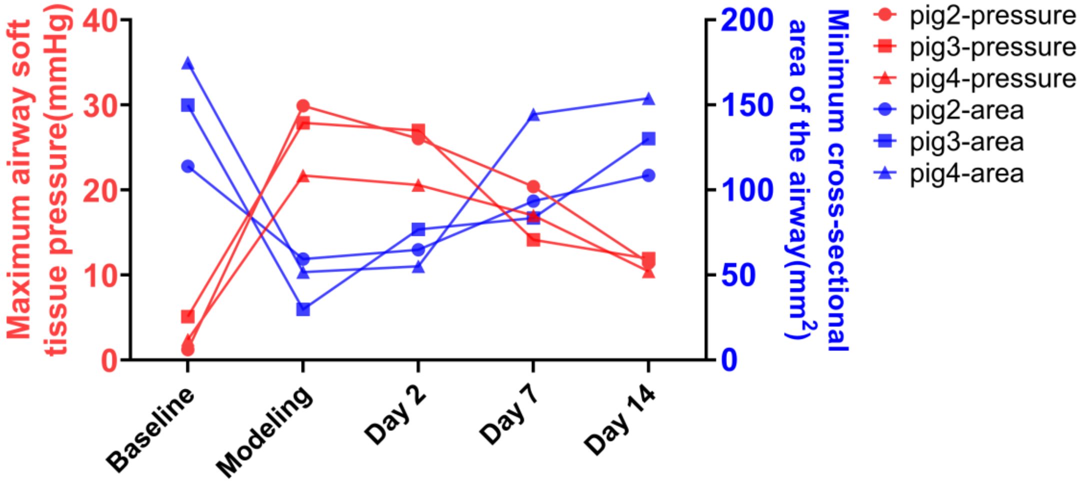

Supplementary Fig. 10. The minimum cross-sectional area of the upper airway and the maximum airway soft tissue pressure during OSA events of the three OSA model pigs in the experimental group at different time points before and after modeling.

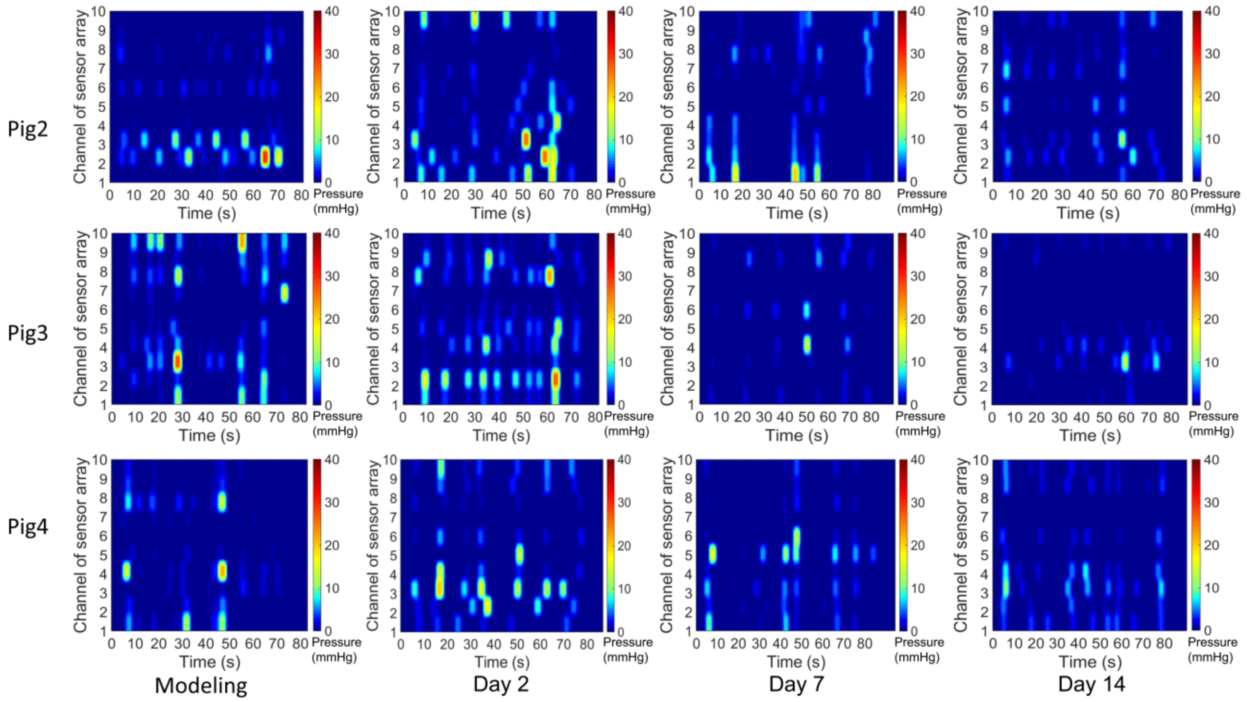

Supplementary Fig. 11. Heatmaps of the airway soft tissue pressure distribution over time for each pig in the experimental group at different time points.

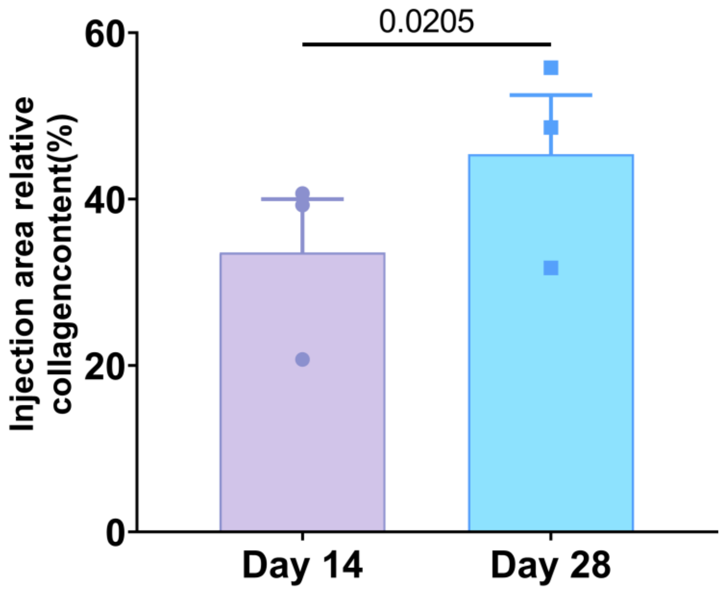

Supplementary Fig. 12. Changes of the relative collagen content at the injection site on days 14 and 28 post-modeling. All sections were compared under the objective X100 parameter, n=3 independent samples. Data are mean ± standard deviation, and comparisons between groups were made using the paired t-test.

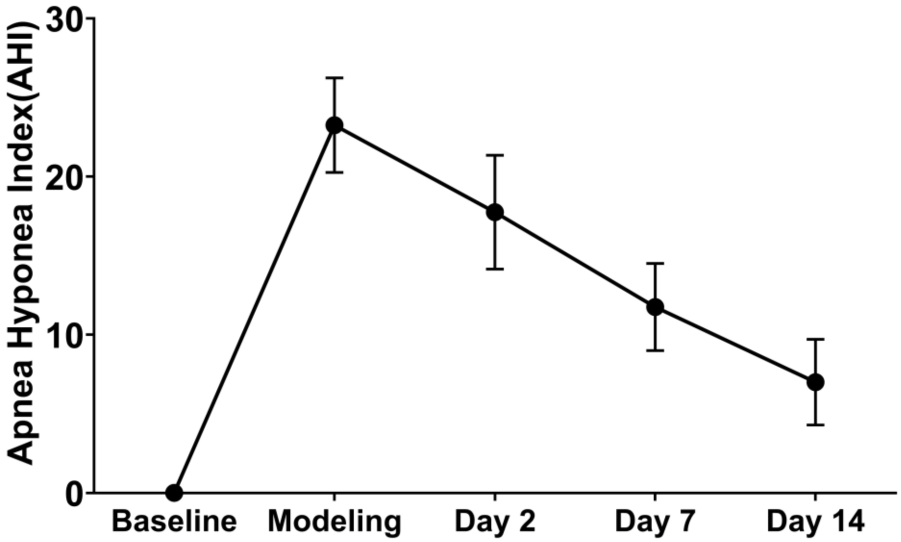

Supplementary Fig. 13. Changes of AHI in OSA model pigs at different time points before and after modeling. Data are mean ± standard deviation, n=4 independent samples.

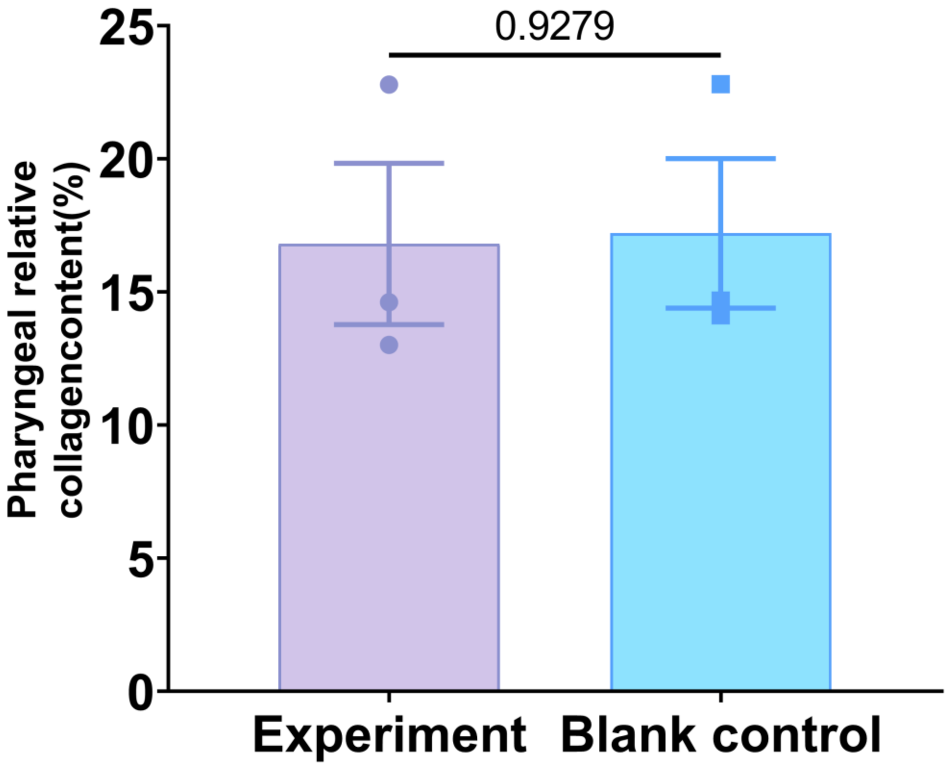

Supplementary Fig. 14. Changes of the relative collagen content at the injection site on days 14 and 28 post-modeling. All sections were compared under the objective X100 paramete, n=3 samples. Data are mean ± standard deviation, and comparisons between groups were made using the independent samples t-test.

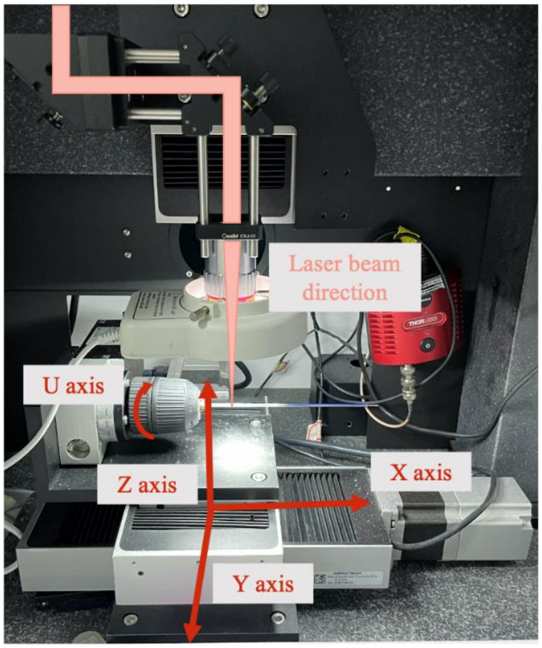

Supplementary Fig. 15. The femtosecond laser processing system with a four-axis displacement platform.

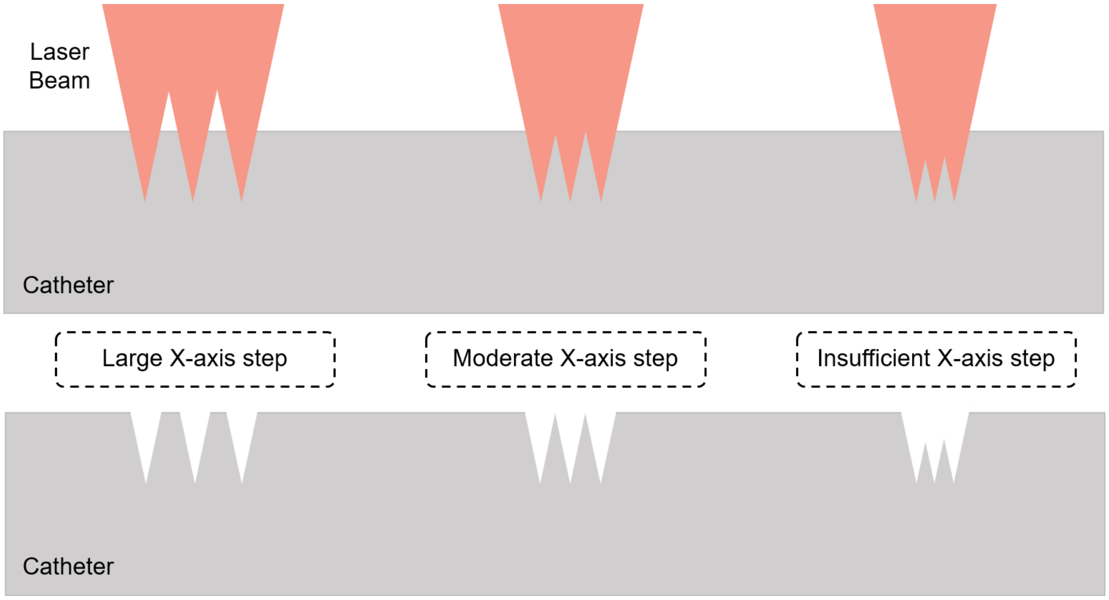

Supplementary Fig. 16. Effect of laser stepping on the surface morphology of microstructures. The step parameter between adjacent nicks significantly influences the shape and density of the microstructure. When the step is excessively large, an unetched section will remain on the tube surface between two nicks, resulting in a trapezoidal microstructure. Conversely, when the step is appropriately set, the two notch edges align to form a conical microstructure with its tip flush against the tube wall. However, if the step is too small, although a conical structure forms due to intersecting scores, its height decreases and the cone tip falls below the outer surface of the catheter. Consequently, this leads to partial loss of the response curve for the initial segment by the sensor.

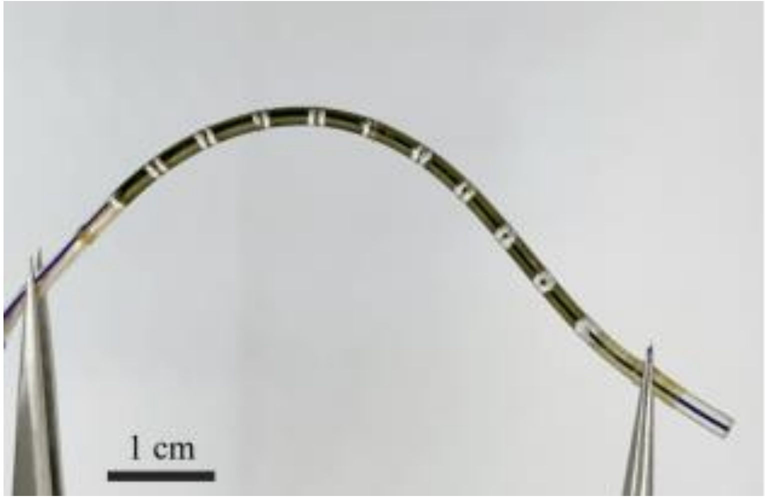

Supplementary Fig. 17. The catheter-based microstructure array with electrodes.

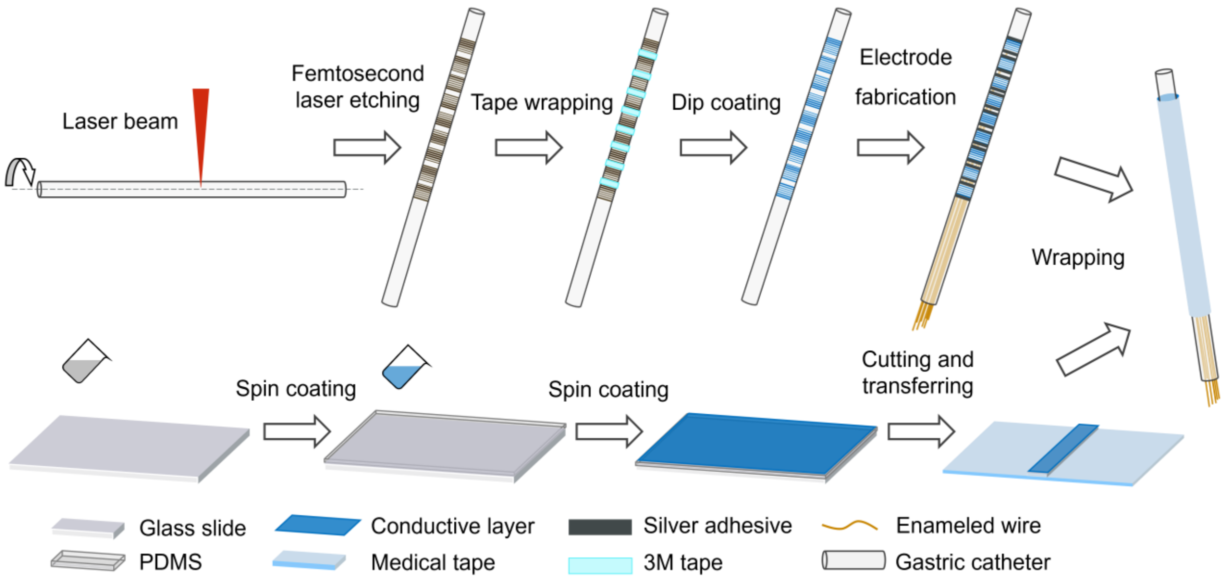

Supplementary Fig. 18. The detailed manufacturing process of the sensor array.

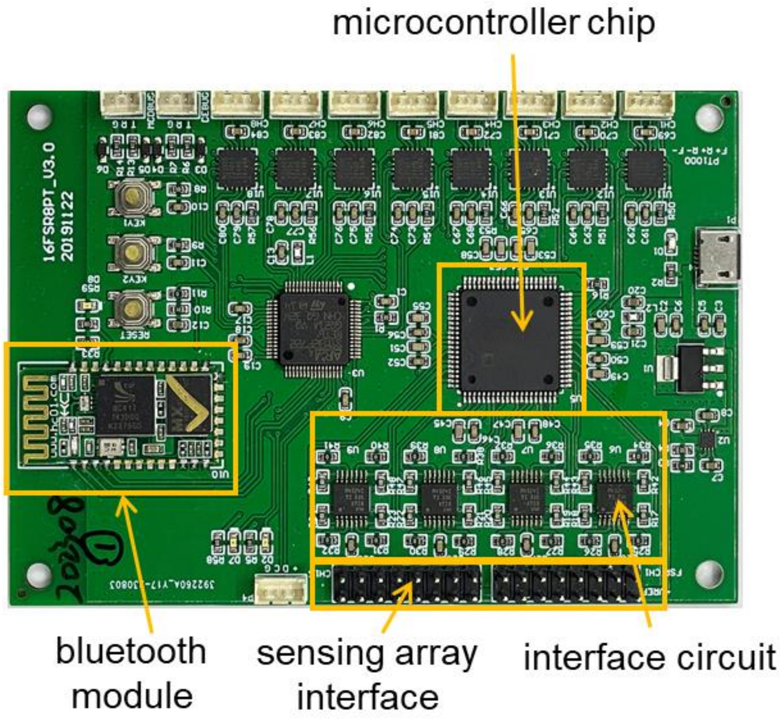

Supplementary Fig. 19. Wireless portable signal acquisition hardware circuit board for the sensor array.

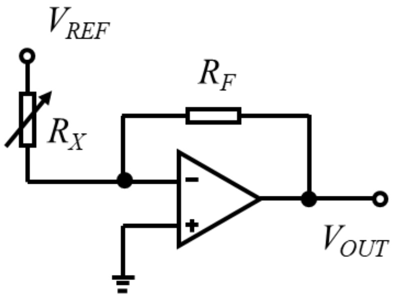

Supplementary Fig. 20. The interface circuit of the hardware board.

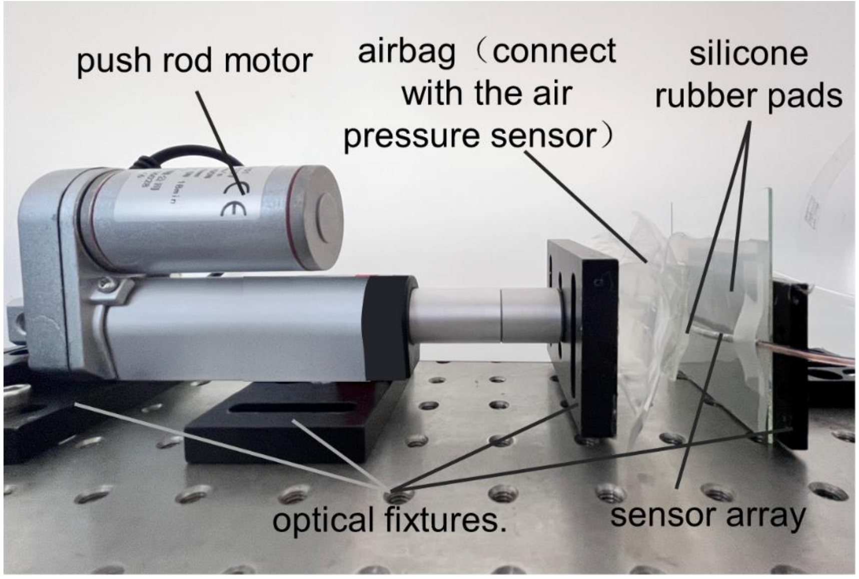

Supplementary Fig. 21. The in vitro test platform for sensor testing.

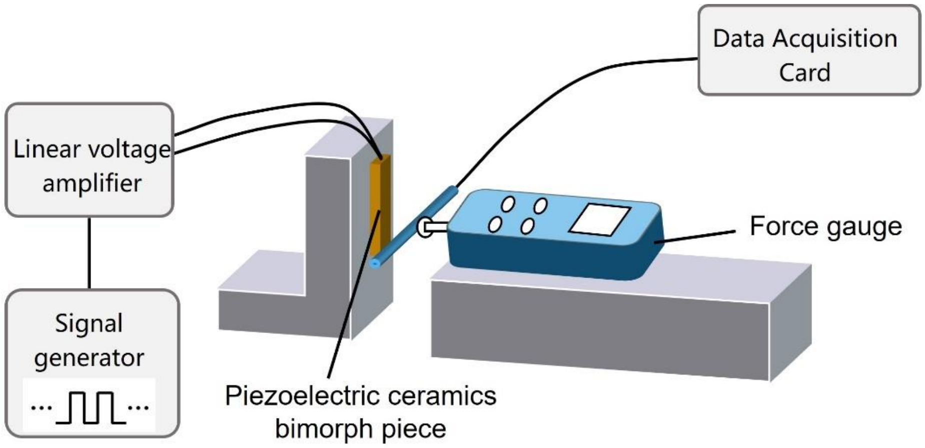

Supplementary Fig. 22. Scenario illustration for sensor sensitivity testing.

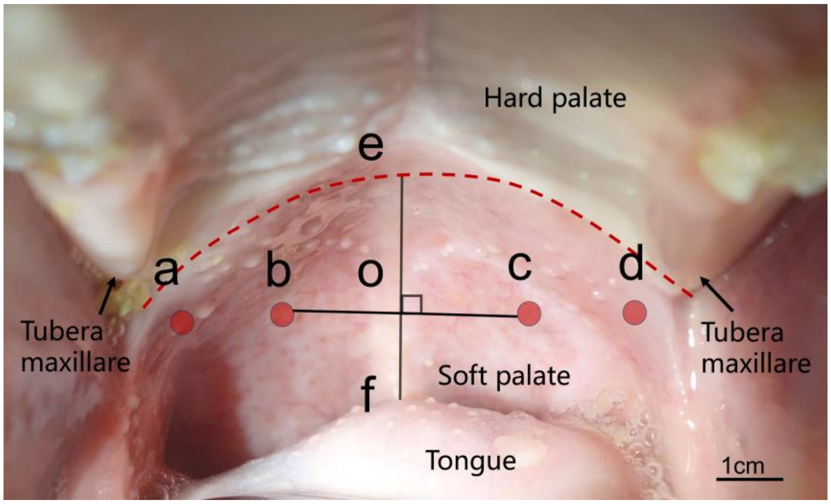

Supplementary Fig. 23. Schematic diagram of injection sites for OSA pig modeling. The injection sites for OSA pig modeling are denoted as *a*, *b*, *c*, and *d*. The line *ef* corresponds to the middle palatal line, with e representing its intersection point with the posterior margin of the hard palate. Perpendicular to *ef* is line *bc* intersecting at point o, where *bo*=*co*=2cm and *eo*=3cm. Injection sites a and d are situated on the lateral pharyngeal wall behind bilateral maxillary nodules along an extended line of *bc*.

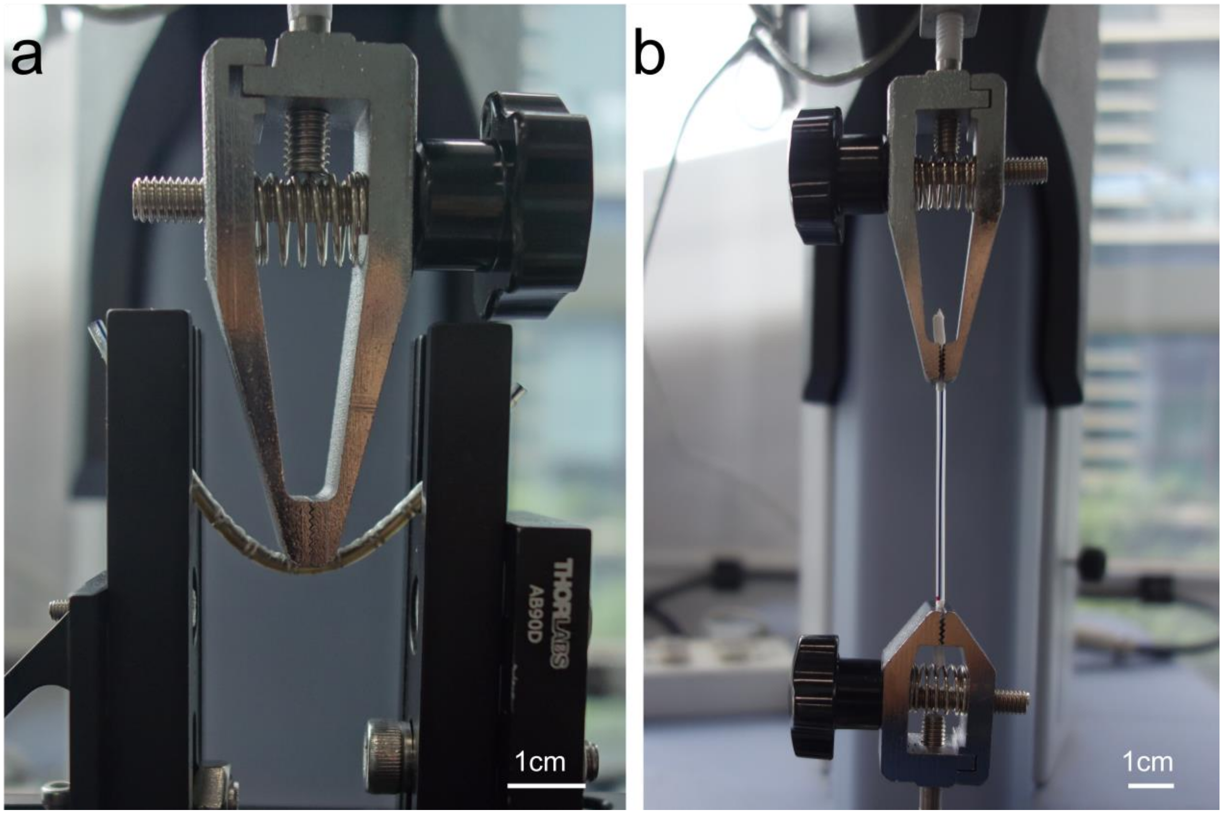

Supplementary Fig. 24. The device bending modulus (a) and tensile modulus(b) test setup.

Supplementary Table 1. Elastic Modulus of clinically used trans-nasal tubing devices

**Item**	**Gastric tube 1**	**Gastric tube 2**	**Gastric tube 3**	**PH-Impedance Catheter**	**Nasal trachea cannula 1**	**Nasal trachea cannula 2**	**Sensor Array**
Material	Silicone	Silicone	Polyurethane	Polyurethane	PVC	PVC	Polyurethane Base + PDMS + Polyurethane Film
Outer Diameter (mm)	2	2	2	2	3	3	2.6
Inner Diameter (mm)	1.2	1.2	1.2	1.4	2	2	1.2
Bending Modulus (MPa)	10.90	4.52	14.21	47.84	39.21	44.83	5.86
Tensile Modulus (MPa)	22.4	6.21	22.17	34.67	87.92	90.06	26.13

Devices manufacturer:
Gastric tube 1: Yangzhou Huayue Technology Development Co., Ltd. HUAYUE Gastric Tube. Yangzhou, China.
Gastric tube 2: Huizhong International Medical Devices Co., Ltd. VREPER Gastric Tube. Beijing, China.
Gastric tube 3: Nutricia Pharmaceutical (Wuxi) Co., Ltd. Flocare Nasogastric Tube. Wuxi, China.
Sensor Array: This work.
PH-Impedance Catheter: Chongqing Jinshan Science & Technology (Group) Co., Ltd. Jinshan pH-Impedance Catheter. Chongqing, China.
Nasal trachea cannula 1: Henan Tuoren Medical Instrument Group Co., Ltd. TUOREN Tracheal Tube. Henan, China.
Nasal trachea cannula 2: Jiangxi Glance Medical Equipment Co., Ltd. GLANCE MEDICAL Tracheal Tube. Jiangxi, China.

Supplementary Table 2 Different solutions for locating OSA obstruction sites

Method	Sedation Required	Invasive/non-invasive	Direct/Indirect Measurement	Device Size	Locating accuracy	Additional safety requirement
**Polysomnography (PSG)**[1-3]	Yes (for some)	Invasive	Indirect	Diameter 2mm (sensor module)	A sensor module is required to determine if the airway is obstructed by comparing air pressure differences.	
**Flexible Laryngoscopy**[4,5]	No	Invasive	Direct	Diameter 3-5mm (laryngoscopy)	Visual examination	Need local anesthetic
**Multi-Sensor Catheters with FBG**[6,7]	Yes (for some)	Invasive	Direct	Diameter about 2-3mm	It can accurately measure the pressure gradient along the airway during sleep but is poorly flexible.	Need local anesthetic (for some)
**Optical Coherence Tomography (OCT)**[8-10]	No	Invasive	Direct	Diameter 1.2-3.8 mm(catheter)	High resolution for anatomy, but accuracy can be compromised by interference from respiratory movements and soft tissue collapse.	
**Drug-Induced Sleep Endoscopy (DISE)**[11-14]	Yes	Invasive	Direct	Diameter 3-5mm (endoscope)	Dynamic imaging during sleep	Need local anesthetic
**Pharyngoesophageal Manometry**[15,16]	No	Invasive	Indirect	Diameter 2-5 mm(catheter)	Low resolution	

**Acoustic Pharyngometry**[17,18]	No	Non-invasive	Indirect	Probe diameter 10-15 mm	It cannot directly measure dynamic airway collapse and only provides anatomical information during static breathing.	
**Rhinometry**[18,19]	No	Non-invasive	Indirect	Probe diameter 10-15 mm	It cannot directly measure dynamic airway collapse and only provides anatomical information during static breathing.	
**Local Ultrasound**[20-23]	No	Non-invasive	Direct	Probe diameter 20-50 mm	Unable to accurately locate airway obstruction in multiple locations	
**Snoring Sound Frequency Analysis**[19-22]	No	Non-invasive	Indirect	N/A	The accuracy can be affected by multiple interfering factors. Unable to accurately locate airway obstruction in multiple locations	
**Flow Pattern Analysis**[28]	No	Non-invasive	Indirect	N/A	Unable to accurately locate airway obstruction in multiple locations	
**Computed Tomography (CT)**[29]	No	Non-invasive	Direct	Scanner	High for anatomy, but no functional information	Ionizing radiation risk
**Cine-MRI**[30-32]	Yes	Non-invasive	Direct	Scanner	Real-time imaging	
**Catheter-Based Sensor Arrays (This work)**	No	Invasive	Direct	Diameter 2.6 mm	It allows direct on-site pressure measurements, localizes obstructions to the millimeter level, and detects airway obstructions in multiple locations simultaneously.	

References

1. Morales Divo, C. et al. Polysomnography and ApneaGraph® in Patients with Sleep-Related Breathing Disorders. *O.R.L. Journal for Oto-Rhino-Laryngology and its Related Specialties*. **71**, 27-31 (2009).
2. Singh, A., Al-Reefy, H., Hewitt, R. & Kotecha, B. Evaluation of ApneaGraph in the diagnosis of sleep-related breathing disorders. *Eur. Arch. Otorhinolaryngol.* **265**, 1489-1494 (2008).
3. Ng, A. T., Qian, J. & Cistulli, P. A. Oropharyngeal collapse predicts treatment response with oral appliance therapy in obstructive sleep apnea. *Sleep*. **29**, 666-671 (2006).
4. Torre, C., Zaghi, S., Camacho, M., Capasso, R. & Liu, S. Y. Hypopharyngeal evaluation in obstructive sleep apnea with awake flexible laryngoscopy: Validation and updates to Cormack-Lehane and Modified Cormack-Lehane scoring systems. *Clin. Otolaryngol.* **43**, 823-827 (2018).
5. Narayanan, A. & Faizal, B. Correlation of Lateral Cephalogram and Flexible Laryngoscopy with Sleep Study in Obstructive Sleep Apnea. *Int J Otolaryngol*. **2015**, 127842 (2015).
6. Cook, P. R. et al. Characterizing collapse during obstructive sleep apnea through fiber optic manometry. 2020 2020-1-1: SPIE; 2020. p. 112330K.
7. Wall, A. J. et al. A multimodal optical catheter for diagnosing obstructive sleep apnea. 2019 2019-1-1: SPIE; 2019. p. 1087203.
8. Jing, J., Zhang, J., Loy, A. C., Wong, B. J. F. & Chen, Z. High-speed upper-airway imaging using full-range optical coherence tomography. *J. Biomed. Opt.* **17**, 110501-110507 (2012).
9. Leigh, M. S. et al. Anatomical Optical Coherence Tomography for Long-Term, Portable, Quantitative Endoscopy. *IEEE. Trans. Biomed. Eng.* **55**, 1438-1446 (2008).
10. Armstrong, J. J. et al. In vivo size and shape measurement of the human upper airway using endoscopic long-range optical coherence tomography. *Opt. Express*. **11**, 1817 (2003).
11. Di Bari, M. et al. The effect of drug-induced sleep endoscopy on surgical outcomes for obstructive sleep apnea: a systematic review. *Sleep Breath.* **28**, 859-867 (2024).
12. Viana, A., Estevao, D. & Zhao, C. The clinical application progress and potential of drug-induced sleep endoscopy in obstructive sleep apnea. *Ann. Med.* **54**, 2909-2920 (2022).
13. Lee, E. J. & Cho, J. H. Meta-Analysis of Obstruction Site Observed With Drug-Induced Sleep Endoscopy in Patients With Obstructive Sleep Apnea. *The Laryngoscope*. **129**, 1235-1243 (2019).
14. Victores, A. J., Olson, K. & Takashima, M. Interventional Drug-Induced Sleep Endoscopy: A Novel Technique to Guide Surgical Planning for Obstructive Sleep Apnea. *J. Clin. Sleep Med.* **13**, 169-174 (2017).
15. Oliveira, L. A., Fontes, L. H. & Cahali, M. B. Swallowing and pharyngo-esophageal manometry in obstructive sleep apnea. *Braz. J. Otorhinolaryngol.* **81**, 294-300 (2015).
16. Reda, M., Gibson, G. J. & Wilson, J. A. Pharyngoesophageal pressure monitoring in sleep apnea syndrome. *Otolaryngol. Head Neck Surg.* **125**, 324-331 (2001).
17. Agarwal, S. S., Datana, S., Roy, I. D. & Andhare, P. Effectiveness of Titratable Oral Appliance in Management of Moderate to Severe Obstructive Sleep Apnea—A Prospective Clinical Study with Acoustic Pharyngometry. *Indian Journal of Otolaryngology, and Head, and Neck Surgery*. **74**, 409-415 (2022).
18. Bokov, P. et al. The utility of acoustic pharyngometry and rhinometry in pediatric obstructive sleep apnea syndrome. *Sleep Med.* **58**, 75-81 (2019).
19. Agarwal, S. S., Datana, S., Sahoo, N. K. & Bhandari, S. K. Correlating Nasal Patency with Obstructive Sleep Apnea in Obese Versus Non-Obese Patients: An Acoustic Rhinometry Study. *Indian Journal of Otolaryngology, and Head, and Neck Surgery*. **74**, 1483-1491 (2022).
20. Singh, M. et al. Point-of-Care Ultrasound for Obstructive Sleep Apnea Screening: Are We There Yet? A Systematic Review and Meta-analysis. *Anesthesia & Analgesia*. **129**, 1673-1691 (2019).
21. Chen, J. W., Huang, C. C., Weng, C. K., Chang, C. H. & Wang, S. J. Simultaneous recording of ultrasound and polysomnography during natural sleep in patients with obstructive sleep apnea: a pilot study. *J. Sleep Res.* **26**, 481-486 (2017).
22. Weng, C. K., Chen, J. W., Lee, P. Y. & Huang, C. C. Implementation of a Wearable Ultrasound Device for the Overnight Monitoring of Tongue Base Deformation during Obstructive Sleep Apnea Events. *Ultrasound Med. Biol.* **43**, 1639-1650 (2017).
23. Weng, C., Chen, J. & Huang, C. A FPGA-based wearable ultrasound device for monitoring obstructive sleep apnea syndrome. 2015 2015-1-1: IEEE; 2015. p. 1-4.
24. Sebastian, A., Cistulli, P. A., Cohen, G. & Chazal, P. D. Characterisation of Upper Airway Collapse in OSA Patients Using Snore Signals: A Cluster Analysis Approach. 2020 2020-1-1: IEEE; 2020. p. 5124-5127.

25. Ding, L. & Peng, J. Automatic classification of snoring sounds from excitation locations based on prototypical network. *Appl. Acoust.* **195**, 108799 (2022).
26. Sun, J., Hu, X., Peng, S., Peng, C. & Ma, Y. Automatic classification of excitation location of snoring sounds. *J. Clin. Sleep Med.* **17**, 1031-1038 (2021).
27. Huang, Z. et al. Prediction of the obstruction sites in the upper airway in sleep-disordered breathing based on snoring sound parameters: a systematic review. *Sleep Med.* **88**, 116-133 (2021).
28. Genta, P. R. et al. Airflow Shape Is Associated With the Pharyngeal Structure Causing OSA. *Chest*. **152**, 537-546 (2017).
29. Mahale, A. R., Rao, P., Ullal, S., Fernandes, M. & Prabhu, S. Computed Tomography and Cephalometric Evaluation of Obstructive Sleep Apnea Syndrome. *Indian Journal of Otolaryngology, and Head, and Neck Surgery*. **74**, 5134-5143 (2022).
30. Li, Y. et al. Characteristics and Mechanism of Upper Airway Collapse Revealed by Dynamic MRI During Natural Sleep in Patients with Severe Obstructive Sleep Apnea. *Nat. Sci. Sleep*. **15**, 885-902 (2023).
31. Volner, K., Chao, S. & Camacho, M. Dynamic sleep MRI in obstructive sleep apnea: a systematic review and meta-analysis. *Eur. Arch. Otorhinolaryngol.* **279**, 595-607 (2022).
32. Kojima, T. et al. Assessment by airway ellipticity on cine-MRI to differentiate severe obstructive sleep apnea. *The Clinical Respiratory Journal*. **12**, 878-884 (2018).
