# Supplementary material for: A flexible catheter-based sensor array for upper airway soft tissues pressure monitoring
Source: Nat Commun. 2025 Jan 2;16:287. doi: 10.1038/s41467-024-55088-y (PMC11695590; doi:10.1038/s41467-024-55088-y)
Supplement: Supplementary file 2 — Description of Additional Supplementary Files [file 41467_2024_55088_MOESM2_ESM.pdf]

Description of Additional Supplementary Files

Supplementary Movie 1: A representative OSA event among three OSA model pigs in the experimental group.
