# Supplementary material for: A flexible catheter-based sensor array for upper airway soft tissues pressure monitoring
Source: Nat Commun. 2025 Jan 2;16:287. doi: 10.1038/s41467-024-55088-y (PMC11695590; doi:10.1038/s41467-024-55088-y)
Supplement: Supplementary file 4 — Reporting Summary [file 41467_2024_55088_MOESM4_ESM.pdf]

nature portfolio

Corresponding author(s): Ruya Li and Yubo Fan

Last updated by author(s): Nov 13, 2024

Reporting Summary

Nature Portfolio wishes to improve the reproducibility of the work that we publish. This form provides structure for consistency and transparency in reporting. For further information on Nature Portfolio policies, see our Editorial Policies and the Editorial Policy Checklist.

Statistics

For all statistical analyses, confirm that the following items are present in the figure legend, table legend, main text, or Methods section.

n/a	Confirmed	
[ ]	[x]	The exact sample size (n) for each experimental group/condition, given as a discrete number and unit of measurement
[ ]	[x]	A statement on whether measurements were taken from distinct samples or whether the same sample was measured repeatedly
[ ]	[x]	The statistical test(s) used AND whether they are one- or two-sided<br>*Only common tests should be described solely by name; describe more complex techniques in the Methods section.*
[x]	[ ]	A description of all covariates tested
[x]	[ ]	A description of any assumptions or corrections, such as tests of normality and adjustment for multiple comparisons
[ ]	[x]	A full description of the statistical parameters including central tendency (e.g. means) or other basic estimates (e.g. regression coefficient) AND variation (e.g. standard deviation) or associated estimates of uncertainty (e.g. confidence intervals)
[ ]	[x]	For null hypothesis testing, the test statistic (e.g. F, t, r) with confidence intervals, effect sizes, degrees of freedom and P value noted<br>*Give P values as exact values whenever suitable.*
[x]	[ ]	For Bayesian analysis, information on the choice of priors and Markov chain Monte Carlo settings
[x]	[ ]	For hierarchical and complex designs, identification of the appropriate level for tests and full reporting of outcomes
[x]	[ ]	Estimates of effect sizes (e.g. Cohen's d, Pearson's r), indicating how they were calculated

Our web collection on statistics for biologists contains articles on many of the points above.

Software and code

Policy information about availability of computer code

Data collection	Signal acquisition for the sensor array was conducted using a wireless, portable acquisition system. The system comprised two main components: a hardware circuit board and PC-based acquisition software. The hardware board featured an interface circuit, a microcontroller chip (STM32F722RET6), and a Bluetooth module, powered by a 5V portable charger.Sleep data were collected using a multi-lead polysomnography (Smote PSG, COMPUMEDICS, Australia).
Data analysis	MATLAB (R2020b);<br>ABAQUS 2022<br>Mimics Medical 21.0 ;<br>SPSS 21.0;<br>ImageJ (version 1.53g);<br>GraphPad Prism 9.3;<br>Origin 2018b;<br>The built-in analysis software (ProFusion PSG4) was used for analysis of PSG data.

For manuscripts utilizing custom algorithms or software that are central to the research but not yet described in published literature, software must be made available to editors and reviewers. We strongly encourage code deposition in a community repository (e.g. GitHub). See the Nature Portfolio guidelines for submitting code & software for further information.

Data

Policy information about availability of data

All manuscripts must include a data availability statement. This statement should provide the following information, where applicable:

- Accession codes, unique identifiers, or web links for publicly available datasets
- A description of any restrictions on data availability
- For clinical datasets or third party data, please ensure that the statement adheres to our policy

The data that support the findings of this study are available from the corresponding authors upon request.

Research involving human participants, their data, or biological material

Policy information about studies with human participants or human data. See also policy information about sex, gender (identity/presentation), and sexual orientation and race, ethnicity and racism.

Reporting on sex and gender	We did not performed experiments on human participants.
Reporting on race, ethnicity, or other socially relevant groupings	N/A
Population characteristics	N/A
Recruitment	N/A
Ethics oversight	N/A

Note that full information on the approval of the study protocol must also be provided in the manuscript.

Field-specific reporting

Please select the one below that is the best fit for your research. If you are not sure, read the appropriate sections before making your selection.

☒ Life sciences ☐ Behavioural & social sciences ☐ Ecological, evolutionary & environmental sciences

For a reference copy of the document with all sections, see nature.com/documents/nr-reporting-summary-flat.pdf

Life sciences study design

All studies must disclose on these points even when the disclosure is negative.

Sample size	Sample size calculations were not performed. In the conductive layer conductivity assay, the sample size was n=15 (consisting of five independent samples with three positions tested per sample). This number of independent replicates was sufficient to determine if the P<0.05, to provide sufficient statistical significantly.The main objective of this study is to demonstrate new devices. We used 3 Bama pigs to study the data from the sensing array.We did not perform statistical difference analysis and therefore did not pre-determine the sample size. When comparing the collagen fiber content of the tissues , we used a sample size of n=3. We considered this sample size to be adequate for animal ethics and the scientific validity of the results.
Data exclusions	No data was excluded.
Replication	Data acquisition was carried out on multiple animals to verify the accuracy of the device. We performed sensor array monitoring in 3 OSA model pigs at pre-modeling, modeling, and 2, 7, 14 days post-modeling. Our replication studies were successful.
Randomization	Randomization was not relevant to our study since experiments were specific to each subject.
Blinding	Blinding was not relevant to our study since experiments were specific to each subject.

Reporting for specific materials, systems and methods

We require information from authors about some types of materials, experimental systems and methods used in many studies. Here, indicate whether each material, system or method listed is relevant to your study. If you are not sure if a list item applies to your research, read the appropriate section before selecting a response.

Materials & experimental systems

n/a	Involved in the study
☒	☐ Antibodies
☒	☐ Eukaryotic cell lines
☒	☐ Palaeontology and archaeology
☐	☒ Animals and other organisms
☒	☐ Clinical data
☒	☐ Dual use research of concern
☒	☐ Plants

Methods

n/a	Involved in the study
☒	☐ ChIP-seq
☒	☐ Flow cytometry
☒	☐ MRI-based neuroimaging

Animals and other research organisms

Policy information about studies involving animals; ARRIVE guidelines recommended for reporting animal research, and Sex and Gender in Research

Laboratory animals	Four male Bama pigs between six to seven months and weighing between 32 and 35 kg were selected for the study.
Wild animals	This study did not involve wild animals.
Reporting on sex	Sex was not considered in the study.
Field-collected samples	This study did not involve samples collected from the field.
Ethics oversight	The experiments were conducted following the protocols approved by the Biomedical Ethics Committee of Beihang University, approval number BM20230259.

Note that full information on the approval of the study protocol must also be provided in the manuscript.

Plants

Seed stocks	The study did not involve plant samples.
Novel plant genotypes	The study did not involve plant samples.
Authentication	The study did not involve plant samples.
